# Behavioral and Psychological Factors Associated with Diabetes Self-Management in Adults with Diabetes

**DOI:** 10.3390/healthcare14142171

**Published:** 2026-07-18

**Authors:** Rasha Mohammed Hussien, Ayman Mohamed El-Ashry, Nora A. AlFaris, Abdelaziz Hendy

**Affiliations:** 1 Department of Community, Psychiatric, and Mental Health Nursing, College of Nursing, Qassim University, Buraydah 52571, Saudi Arabia; rm.ahmed@qu.edu.sa; 2 Psychiatric Mental Health Nursing, Faculty of Nursing, Alexandria University, Alexandria 21511, Egypt; 3 Department of Sports Health, College of Sports Sciences & Physical Activity, Princess Nourah bint Abdulrahman University, Riyadh 11671, Saudi Arabia; 4 Department of Maternal and Child Health Nursing, College of Nursing, Qassim University, Buraydah 52571, Saudi Arabia

**Keywords:** diabetes mellitus, self-management, fear of hypoglycemia, HbA1c, psychological factors, glycemic control

## Abstract

Background: Diabetes self-management (DSM) is essential for optimal glycemic control and prevention of complications. Psychological factors, particularly fear of hypoglycemia (FOH), may be associated with self-management behaviors, yet their relationship with glycemic outcomes remains unclear. Objective: To examine the associations between FOH, DSM, and glycemic control (HbA1c) and to identify demographic and clinical factors associated with DSM among adults with diabetes. Methods: A cross-sectional descriptive correlational study was conducted among 180 adults attending outpatient clinics at Qassim University Medical City between October 2025 and January 2026. Data were collected using a structured questionnaire including demographic and clinical variables, the Diabetes Self-Management Questionnaire (DSMQ), and a validated FOH screening tool. Glycemic control was assessed using the most recent HbA1c value documented in the medical record within the previous three months. Pearson’s correlation and multiple linear regression analyses were performed using SPSS version 26. Results: The mean DSM score was 26.71 ± 3.57, indicating a moderately acceptable level, with physical activity as the lowest-performing domain. The mean HbA1c was 7.95 ± 1.85%, reflecting suboptimal control. FOH was positively associated with DSM (r = 0.49, *p* < 0.001) but not with HbA1c. DSM was not significantly correlated with HbA1c. In the regression analysis, FOH showed the strongest independent association with DSM (β = 0.44, *p* < 0.001). The model explained 50.1% of the variance. Conclusions: FOH was significantly associated with DSM; however, the cross-sectional design does not allow conclusions regarding whether this association reflects adaptive concern, maladaptive fear, or greater self-care awareness. The lack of association between FOH, DSM, and HbA1c highlights the complexity of glycemic control and supports the need for integrated, patient-centered interventions addressing both behavioral and psychological aspects of diabetes care.

## 1. Introduction

Elevated blood glucose levels are an indicator of diabetes mellitus (DM), a chronic condition that can damage the nerves, vascular system, and other organs. This damage is caused by either insufficient insulin production or the body’s failure to use insulin properly [1]. DM may affect individuals across all age groups, genders, and geographical locations and is a leading cause of morbidity and death worldwide. Environmental factors combined with genetic predisposition account for over 90% of type 2 diabetes cases [2].

DM is a major global health concern, ranked among the most serious chronic conditions due to its high prevalence and serious complications [3]. The updated report by the International Diabetes Federation (IDF) indicates that by 2050, the number of individuals with diabetes will reach 853 million, accounting for approximately 1 in 8 adults, representing a 46% increase. Saudi Arabia is one of the top ten countries with the highest prevalence of DM in individuals aged 20 to 79. By 2024, approximately 5.3 million people are projected to be living with this condition, and this number is projected to increase to an estimated 9.5 million by 2050 [4,5].

A previous study estimated that complications related to diabetes caused approximately 32,000 deaths [6]. Those with uncontrolled diabetes are more vulnerable to experiencing ramifications such as neuropathy, foot ulcers, cerebrovascular diseases, renal failure, myocardial infarction, and blindness. Furthermore, DM was found to be the primary cause of disability in Saudi Arabia [7]. DM patients need to be empowered with the necessary knowledge and skills to participate in self-care management [8], which calls for new strategies to improve patient education and management [9].

Diabetes self-management is associated with improved health outcomes and quality of life [10]. Diabetes self-management (DSM) requires adherence to medication regimens and lifestyle behaviors, including physical activity, dietary modifications, self-monitoring, consistent medical appointments, and the management of psychosocial stressors [11], alongside foot care, smoking cessation, and the self-monitoring of blood glucose (SMBG) and HbA1c levels [12]. Integrating these actions into daily routines often proves difficult [13], which can subsequently impede individuals’ capacity to manage their diabetes effectively and comply with recommended self-care regimens [14]. Consequently, it is imperative to identify potential barriers to effective self-management and address the underlying mechanisms to inform the development of interventions that promote self-management.

Managing diabetes is a complex, lifelong process, and maintaining self-management behavior can be challenging [15]. Patients with diabetes face multiple barriers that hinder access to quality care. These include logistic challenges such as difficulty reaching appointments promptly, particularly in rural areas [16]. Cultural considerations exert a significant influence on diabetes management and the mitigation of HbA1c levels, particularly when intertwined with sedentary lifestyles and non-medical determinants such as concerns about medication side effects (20.4%) and the complexities of polypharmacy (19.1%) [17]. Moreover, insufficient patient education remains a significant factor in suboptimal self-management, particularly regarding medication adherence and its adverse effects [18].

The goal of diabetic care and treatment is to maintain metabolic control and reduce the risk of developing microvascular and macrovascular complications [18]. To accomplish this goal, DM patients must be compliant with insulin or hypoglycemic medications and modify their lifestyles. Nevertheless, the therapeutic measures essential to attain glycemic control may lead to vulnerability to hypoglycemia [19]. A blood glucose level below 70 mg/dL is referred to as hypoglycemia. Hypoglycemia can be classified into three levels: Level 1, defined as a blood glucose level below 70 mg/dL but at least 54 mg/dL; Level 2, defined as a blood glucose level below 54 mg/dL; and Level 3, defined as a severe hypoglycemic event requiring third-party assistance for recovery, regardless of the measured glucose value [20]. Recurrent hypoglycemia is linked with cognitive impairment, hippocampal damage, neuropathy, and an increased long-term risk of dementia [21].

Repeated episodes of hypoglycemia that interfere with activities of daily living can lead to avoidance behaviors that impede optimal glycemic control and reduce quality of life, thereby causing psychological distress and the development of fear of hypoglycemia (FOH). FOH is described as a generalized anxiety disorder marked by a disproportionate anticipatory response to a perceived threat that is initially real and causes the patient extreme uncertainty and distress [22].

Earlier research evidence indicated that FOH is more likely to be higher in type 1 diabetes, an insulin-dependent form, than in type 2 diabetes (T2D) [23]. However, research on FoH in T2D patients has grown steadily, driven by the high prevalence of T2D, the popularity of intensive glycemic control, and the growing prominence of psychological issues in these patients [24].

Hypoglycemia may lead to dizziness, fainting, and a feeling of impending death [25]. Thus, the patients may exhibit “overcompensatory behaviors,” such as extremely meticulous blood glucose monitoring, restricting physical activity, intentionally lowering the necessary medication dosage, or consuming large quantities of carbohydrates to maintain elevated blood glucose levels [26]. However, FOH represents a significant barrier to blood sugar control, potentially causing or worsening complications in patients and adversely affecting diabetes management [27].

Achieving optimal glycemic control remains a major challenge in Saudi Arabia, where diabetes prevalence and diabetes-related complications continue to impose a substantial clinical burden. Although diabetes self-management education and glycemic monitoring are central to care, many patients continue to experience suboptimal control and difficulty sustaining self-care behaviors. FOH is particularly relevant in this context because it may influence medication adherence, glucose monitoring, dietary choices, and physical activity. However, its behavioral effect may differ across patients: in some individuals, FOH may promote vigilance and preventive self-care, whereas in others it may lead to avoidance behaviors, intentional maintenance of higher glucose levels, or reluctance to intensify treatment.

Both type 1 and type 2 diabetes were included because FOH is clinically relevant in both groups, particularly among patients receiving insulin or hypoglycemia-associated therapies. Including both groups allowed the study to examine FOH and DSM across a heterogeneous outpatient diabetes population; however, this heterogeneity was acknowledged as a limitation. Although previous studies have examined DSM and FOH separately, limited evidence is available regarding how FOH, DSM, HbA1c, and demographic and clinical factors are interrelated among adults with diabetes in Saudi Arabia. Clarifying these associations may help identify patient groups who require more individualized educational and psychological support.

### 1.1. Aim of the Study

This study aimed to examine the associations among fear of hypoglycemia, diabetes self-management, HbA1c, and selected demographic and clinical factors among adults with diabetes.

### 1.2. Research Questions

1. What is the relationship between fear of hypoglycemia and diabetes self-management?
2. Is there an association between diabetes self-management and HbA1c levels?
3. Which demographic and clinical factors are associated with diabetes self-management?

## 2. Methods

### 2.1. Study Design

In accordance with the STROBE guidelines, this study employed a cross-sectional, descriptive, and correlational design to investigate the relationships among the study variables. This design is suitable for examining target associations and identifying key predictors without manipulating the research environment. This study also provides valuable insights that can inform therapeutic strategies and future research.

### 2.2. Setting

The research was conducted at the Abdullah Alothaim Center for Diabetes and outpatient clinics for general medicine, family medicine, and endocrinology at Medical City, Qassim University Hospital, Buraydah, Saudi Arabia. These institutions offer specialized outpatient services, providing comprehensive evidence-based care to patients with DM and other chronic and endocrine medical conditions. Data were collected from October 2025 to January 2026.

### 2.3. Participants

A consecutive non-probability sampling approach was used. Eligible adults with DM who attended the selected outpatient clinics for scheduled follow-up visits during the data-collection period were approached and invited to participate consecutively until the required sample size was achieved.

This sampling method was preferred because of practical considerations, such as time constraints and the limited availability of potential contributors. Although efforts were made to include participants with varied demographic and clinical characteristics, the consecutive non-probability sampling approach from selected outpatient clinics may still have introduced selection bias. Therefore, the findings should be interpreted in relation to this clinical sample and should not be assumed to represent the wider diabetes population. The eligibility criteria included patients with diabetes mellitus (types 1 and 2) with a disease duration exceeding one year, aged 18 years or older, capable of effective communication, willing to participate, and having provided informed consent. Both type 1 and type 2 diabetes were included because FOH is clinically relevant across diabetes types, particularly among patients receiving insulin or other therapies associated with hypoglycemia. The study aimed to examine FOH and DSM in a heterogeneous adult outpatient diabetes population rather than in a single diabetes subtype. To account partially for clinical heterogeneity, diabetes type and treatment type were included as covariates in the multivariable regression model.

Patients with severe cognitive impairment or psychiatric disorders that could interfere with understanding or completing the survey, those requiring immediate hospitalization due to severe complications or acute medical emergencies, individuals who refused to participate in the study, and those unable to provide informed consent were excluded from this study.

### 2.4. Sample Size

The sample size was calculated for multiple linear regression analysis using an anticipated medium effect size (f^2^ = 0.15), significance level of 0.05, statistical power of 80%, and 14 predictors. The minimum required sample size was 135. A total of 180 participants were included to compensate for possible incomplete responses.

### 2.5. Data Collection

Part I: Personal and clinical data section: The questionnaire gathered data on age, sex, marital status, education, living conditions, smoking habits, previous DM counseling sessions, regular exercise routines, treatment duration, duration of DM, presence of hypertension, high cholesterol, type of DM, and type of treatment, as well as height and weight.

The Body Mass Index (BMI) for each patient was computed using the Centers for Disease Control and Prevention (CDC) classification. Height was measured in centimeters and converted to meters for BMI calculation. BMI was calculated as weight in kilograms divided by height in meters squared (kg/m^2^). The participants were classified into four categories based on their BMI: underweight (<18.5 kg/m^2^), normal weight (18.5–24.9 kg/m^2^), overweight (25.0–29.9 kg/m^2^), and obese (≥30.0 kg/m^2^). These classifications were based on universally recognized BMI cutoffs [5].

Part II: The American Diabetes Association’s guidance delineates reasonable glycemic control as HbA1c values below 7% (<53 mmol/mol), whereas inadequate glycemic control is characterized by HbA1c levels of 7.0% or higher (≥53 mmol/mol) [20]. HbA1c values were obtained from participants’ medical records. The most recent HbA1c result documented within the three months before questionnaire completion was used to reflect recent glycemic control. If more than one HbA1c value was available within this period, the value closest to the date of questionnaire completion was recorded.

Part III: Diabetes self-management questionnaire—DSMQ

The 16 DSMQ items address various aspects of diabetes self-management. The items provide a comprehensive overview of various behaviors associated with diabetes self-care. These behaviors are divided into five primary domains: medication adherence, dietary management, blood glucose monitoring, physical activity, and communication with health care providers. Items 1, 4, 6, 10, and 12 were classified as “glucose management,” whereas items 2, 5, 9, and 13 were designated as “dietary control.” Items 8, 11, and 15 were designated as “physical activity,” and items 3, 7, and 14 were designated as “healthcare usage.” Item 16 is intended for inclusion only in the “sum scale” and inquiries about a general evaluation of self-care [28].

Patients rate the degree to which each description corresponds to their experiences over the past eight weeks using a four-point Likert scale that ranges from 0 to 3, with 0 meaning “does not apply to me,” 1 meaning “applies to me to a certain extent,” 2 meaning “applies to me to a considerable extent,” and 3 meaning “applies to me extensively.” This yields an overall DSMQ score ranging from 0 to 48 [28].

Reverse scoring of negatively keyed items was essential to ensure that higher scores accurately indicated better self-management behaviors. Therefore, the item scores were transformed. Adherence to diabetic self-management activities was categorized into three levels based on DMSQ scores converted to a 0–10 scale: insufficient (score < 5), moderately acceptable (score 5–8), and optimal (score > 8) [29]. DSMQ has been validated, translated into many languages (including Arabic), and used in Saudi Arabia [30]. A Cronbach’s alpha of 0.84 indicates sufficient internal consistency, suggesting that the DSMQ is a reliable and credible instrument for assessing self-management behaviors among DM patients in Saudi Arabia [17].

Part IV: Fear-of-hypoglycemia screening

The Fear-of-Hypoglycemia Screener, developed by Liu et al. [31], assesses the level of fear associated with hypoglycemia. It consists of nine items distributed across two domains: worry (six items) and avoidance behavior (three items). Each item is rated on a five-point Likert scale ranging from strongly disagree (1) to strongly agree (5), resulting in a total score ranging from 9 to 45. Higher scores indicate a greater fear of hypoglycemia.

Regarding validity and reliability, the original version underwent exploratory factor analysis (EFA), which identified a single-factor structure explaining 66% of the variance, indicating good construct validity. Confirmatory factor analysis (CFA) further supported this one-factor model, with satisfactory fit indices (CFI = 0.95, TLI = 0.94, RMSEA = 0.06). The scale also demonstrated high internal consistency, with a Cronbach’s alpha of 0.88. In a previous study conducted among 300 Arabic-speaking patients, the scale demonstrated even higher internal consistency, with a Cronbach’s alpha of 0.97 [32].

### 2.6. Pilot Study

After obtaining formal approval, we obtained consent from the relevant authorities. Ten percent of eligible patients participated in a pilot study to assess whether the study tools were reasonable, practical, suitable, and objective. Five experts in medical–surgical, mental health, and psychiatric nursing evaluated the Arabic versions of the instruments for cultural relevance, content accuracy, thoroughness, and item clarity, with special attention to the Saudi culture. It was estimated that participants would need approximately 10 to 15 min to complete the questionnaire.

### 2.7. Study Procedure

Data collection was performed in the previously outlined settings after obtaining administrative approval. A certified counselor invited each patient for an interview to determine eligibility, clarify any questions, and ensure impartial replies. Patients were informed of the study’s importance and objectives before obtaining informed consent. Patients completed the questionnaire independently, and the completed forms were collected promptly to maximize response rates and preserve data integrity.

### 2.8. Statistical Analysis

IBM SPSS Statistics for Windows (Version 26.0; IBM Corp., Armonk, NY, USA) was used to analyze the data. Descriptive statistics were computed for all variables included in the study. Categorical variables were described using frequencies and percentages, whereas continuous variables were described using means and standard deviations, along with the range for certain variables. Pearson’s correlation coefficient was computed to understand the relationship between variables: self-management and glycemic control (HbA1c). The collected datasets were reviewed for completeness before analysis. No missing values were identified for the study variables; therefore, no imputation procedures were required, and all participants were included in the statistical analyses.

Before running the inferential statistics, the assumptions for linear regression were checked for linearity, normality of residuals, homoscedasticity, and multicollinearity. To assess for normality, skewness and kurtosis were computed for the variables included in the model. In addition, the histogram and Q-Q plot were checked for normality of the data. Similarly, the presence of multicollinearity was checked by computing the variance inflation factors (VIF).

A standard multivariable (forced-entry) linear regression model was conducted to identify independent predictors of diabetes self-management. Categorical variables included in the multiple linear regression model were entered as dummy variables. For binary variables, the reference category was coded as 1, while the comparison category was coded as 0. The reference categories used in the regression model were counseling (Yes), hypertension (Yes), hypercholesterolemia (Yes), living alone (Yes), regular exercise (Yes), and type of diabetes (Type 1). For categorical variables with more than two categories, dummy coding was performed using the selected reference categories: treatment duration (5–10 years), duration of diabetes (5–10 years), marital status (Married), education level (bachelor’s degree), and treatment type (combined therapy). Regression coefficients for categorical predictors represent the adjusted difference in diabetes self-management scores associated with the coded comparison category (0) relative to the reference category (1), while controlling for other variables included in the model.

This modeling strategy was selected to estimate the independent effect of fear of hypoglycemia while controlling for potential confounding from all other covariates, rather than prioritizing variable selection.

Regression coefficients (Bs), standard errors (SEs), standardized beta coefficients (βs), t-statistics, *p*-values, and 95% confidence intervals were reported. The model fit was evaluated using R^2^, adjusted R^2^, and the overall F-test. The effect size was calculated using Cohen’s f^2^. Statistical significance was set at *p* < 0.05.

The internal consistency reliability of the main study instruments was assessed using Cronbach’s alpha. The diabetes self-management scale demonstrated high reliability (α = 0.87), as did the fear-of-hypoglycemia screener (α = 0.791).

## 3. Result

Table 1 summarizes the demographic, clinical, and anthropometric characteristics of the study participants (N = 180). The mean age of the participants was 36.82 ± 16.34 years. Most participants were female (64.4%), single (50.0%), and had a bachelor’s degree (43.9%). Most participants lived with others (93.3%) and were non-smokers (88.3%). Regarding clinical characteristics, 35.0% had previously attended diabetes counseling sessions, and 40.0% reported regular exercise. Nearly half had a treatment duration of less than five years (47.8%) and a diabetes duration of less than five years (43.9%). Hypertension and high cholesterol levels were present in 37.8% and 39.4% of the patients, respectively. Type 1 diabetes was reported by 70.0% of the participants. The mean height was 160.86 ± 13.92 cm, and the mean weight was 73.18 ± 14.60 kg. Exclusive insulin therapy was the most common treatment (58.9%).

The mean (SD) self-management score was 26.71 (3.57), with a range of 14–36. The mean and SD of HbA1c levels were 7.95 (1.85), with a range of 5.0–20.0. The Fear-of-Hypoglycemia screening mean score was 26.10 (6.69), as shown in Table 2.

Fear of hypoglycemia was positively and significantly correlated with diabetes self-management (r = 0.50, *p* < 0.001), implying that the fear of hypoglycemia is positively related to diabetes self-management. However, fear of hypoglycemia was not significantly correlated with HbA1c (r = 0.04, *p* > 0.05). The relationship between self-management and HbA1c was negative but not statistically significant (r = −0.13, *p* = 0.087; Table 3).

Fear of hypoglycemia was the strongest associated factor with self-management in the multivariable regression model (β = 0.44, *p* < 0.001). Counseling was also found to have a positive relationship with self-management (β = 0.25, *p* = 0.001), whereas hypertension (β = −0.27, *p* < 0.001), living alone (β = −0.20, *p* = 0.010), and age (β = −0.16, *p* = 0.006) were negatively related to self-management. Education level was found to have a weak positive relationship (*p* = 0.042). The regression model accounted for 50.1% of the variance in self-management (R^2^ = 0.501), as shown in Table 4.

## 4. Discussion

The present study investigated diabetes self-management in adults with diabetes, focusing on fear of hypoglycemia, HbA1c, and some selected demographic and clinical factors. The overall results of the participants indicated moderate self-management abilities, with physical activity being the least acceptable self-management subscale. The mean HbA1c level was higher than the recommended level, indicating suboptimal glycemic control in the study population. Hypoglycemia fears were positively correlated with diabetes self-management and were the strongest correlation in the regression model. Previous diabetes counseling was also positively correlated with self-management, whereas older age, hypertension, and living alone were negatively correlated with self-management. There was a slight positive correlation with education. In contrast, diabetes self-management and fear of hypoglycemia were not significantly correlated with HbA1c. Due to the cross-sectional design of the study, the findings should be interpreted with caution, as they represent only associations.

The moderately acceptable self-management score suggests that individuals engaged in some diabetes-related self-care behaviors, but not at an optimal level. The multidimensionality of diabetes self-management, encompassing adherence to medication, nutritional management, self-monitoring of blood glucose, physical activity, and healthcare utilization, was reflected in this finding [28,29]. Of these domains, physical activity had the lowest score. This finding is clinically relevant, as exercise is an important part of the diabetes regimen, but it is difficult to maintain in everyday life. Multiple sociocultural and environmental barriers may contribute to the low physical activity score observed in the studied population. Factors that may affect physical activity in Saudi Arabia include the influence of a sedentary lifestyle, a hot climate, lack of convenient and culturally accepted space for physical activity, family and work obligations, and fewer opportunities for regular structured activity [12,17]. Other barriers to diabetes self-management reported include time constraints, lifestyle barriers, lack of education, psychological distress, and the inability to include self-care behaviors in their daily routines [13,15].

Additionally, the fact that most of the participants in the present sample were female may be relevant, since gender-related social expectations and responsibilities, as well as preferences for privacy and women-only exercise settings, could affect participation in physical activity. Furthermore, some insulin-treated patients may not be encouraged to engage in exercise due to fear of hypoglycemic episodes during exercise, especially those with type 1 diabetes or insulin therapy [22,27]. These sociocultural and environmental barriers were not measured directly in the present study and should thus be considered as contextual explanations for physical activity behavior and not as confirmed predictors.

Fear of hypoglycemia was positively correlated with diabetes self-management and remained the strongest independently associated factor in the multivariable regression model. This finding should be interpreted cautiously. Patients with greater concern about hypoglycemia may engage more frequently in preventive self-care behaviors, such as blood glucose monitoring, meal planning, medication adherence, and preparation for potential hypoglycemic episodes. Consistent with this interpretation, Ahola et al. [33] reported associations between fear of hypoglycemia and selected self-management behaviors among adults with type 1 diabetes.

However, fear of hypoglycemia may also be associated with maladaptive behaviors, including avoidance of physical activity, unnecessary carbohydrate consumption, intentional maintenance of higher glucose levels, reduction or omission of insulin doses, and reluctance to intensify treatment [22,27]. Because the present study analyzed only the total FOH score, it was not possible to determine whether the observed positive association reflected adaptive preventive behaviors, maladaptive avoidance, or greater awareness of hypoglycemia risk. Therefore, clinicians may consider evaluating the specific behaviors associated with patients’ fear rather than relying solely on the overall level of FOH.

The clinical profile of the sample should also be considered when interpreting this association, as 70.0% of participants had type 1 diabetes and 58.9% received exclusive insulin therapy. FOH may be particularly relevant in these groups because of their greater exposure to hypoglycemia risk [19,23,33]. Although diabetes type and treatment type were included as covariates in the regression model, the study was not sufficiently powered for meaningful subgroup comparisons. Future studies with larger and more balanced samples should examine whether the association between FOH and diabetes self-management differs according to diabetes type, treatment regimen, and specific FOH dimensions.

Neither FOH nor diabetes self-management was significantly associated with HbA1c. One possible explanation is that self-management was only moderately acceptable, with physical activity representing the weakest domain. Thus, the reported self-care behaviors may not have been sufficiently frequent, intensive, or consistent to produce measurable differences in HbA1c. Glycemic control is also influenced by multiple clinical and biological factors, including treatment regimen, insulin dose adjustment, diabetes duration, comorbidities, biological variability, and short-term glucose fluctuations [10,12,20]. In addition, the timing of measurement may have contributed to the findings because self-management was assessed through self-reported recent behaviors, whereas HbA1c reflected glycemic control over the preceding months [20].

Prior diabetes counseling was positively associated with diabetes self-management, corroborating findings that advocate for constructive and engaged counseling and formal diabetes self-management education [9,34]. Nonetheless, very few participants had engaged with counseling. This finding suggests limited access to formal education resources. Counseling may help recognize individual barriers and strengthen adaptive self-care and realistic goal-setting skills.

Diabetes self-management was negatively associated with aging, living alone, and hypertension. In older adults, these may include functional, fatigue, sight and/or memory or medication issues, multiple chronic conditions, and confidence with managing complex treatment [11]. Living alone may indicate a lack of practical and emotional support for medication, meals, glucose self-monitoring, and the management of hypoglycemia. This finding agrees with the reported link between social support and diabetes education and self-management [35]. The negative association with hypertension may be related to the extensive treatment burden of managing multiple chronic conditions that include complex medication and diet regimens, frequent clinic attendance, and increased fatigue [36]. Education was very weakly positively associated with diabetes self-management. This is in agreement with studies that show that education and the understanding of health and diabetes positively influence diabetes self-care [37]. However, this association is weak; thus, formal education may not suffice alone. Emotional well-being, treatment burden, social support, and access to care and practical skills and individualized education may be additional factors that influence diabetes self-management.

Longitudinal studies will be useful in assessing the relationships over time of the psychological variables, diabetes self-management, hypoglycemic events, and glycemic outcomes. Future studies should include multiple measures and assess FOH-related behaviors separately in addition to measuring total FOH scores. Other influences such as nutrition, exercise, obesity, insulin resistance, metabolic control, and other comorbidities associated with diabetes should be studied [38,39]. The present findings stress the need for an integrated and individualized approach to the management of diabetes incorporating behavioral, psychological, clinical, educational and social elements. The findings also stress the importance of recognizing the limitations of the cross-sectional research and the absence of inferential research on the diabetes management approach.

## 5. Conclusions

The study participants demonstrated a moderately acceptable level of diabetes self-management among adults with diabetes, with physical activity identified as the weakest self-management domain. Fear of hypoglycemia was positively associated with diabetes self-management and was the strongest associated factor in the regression model. Previous diabetes counseling was also positively associated with self-management, whereas older age, hypertension, and living alone were negatively associated with self-management. Education showed a weak positive association.

In contrast, diabetes self-management and fear of hypoglycemia were not significantly associated with HbA1c in this sample. These findings suggest that self-management should be interpreted alongside psychological, educational, clinical, and social factors, rather than through glycemic outcomes alone. Because of the cross-sectional design, the findings should be understood as associations and not as evidence of causal relationships.

### 5.1. Limitations

Several limitations should be considered. First, the cross-sectional design limits causal interpretation and does not allow determination of whether FOH leads to better self-management or whether more engaged patients report greater awareness of hypoglycemia risk. Second, participants were recruited using a consecutive non-probability sampling approach from selected outpatient/tertiary-care clinics. This recruitment strategy may have introduced selection bias, as patients attending these clinics may differ from patients managed in primary-care, community, or non-specialized settings. Therefore, the findings should not be generalized to the wider diabetes population without caution. Third, the sample included both type 1 and type 2 diabetes, but participants with type 1 diabetes represented the majority of the sample. Because FOH is usually more common and clinically different among patients with type 1 diabetes and insulin-treated patients, combining diabetes types may have influenced the observed associations. Although diabetes type and treatment type were included as covariates in the regression model, the study was not primarily powered to conduct robust subgroup analyses by diabetes type. Therefore, the findings should be interpreted cautiously and should not be assumed to apply equally to type 1 and type 2 diabetes populations. Fourth, FOH and self-management were assessed using self-report instruments, which may be affected by recall bias and social desirability bias. Fifth, the study did not distinguish between mild, moderate, and severe hypoglycemic episodes, which may have different psychological and behavioral effects. Sixth, glycemic control was evaluated using a single HbA1c measurement, which may not capture short-term glycemic fluctuations or acute behavioral changes. Finally, continuous glucose monitoring data were not available; therefore, glycemic variability, time in range, and frequency of hypoglycemic episodes could not be examined. Although the FOH screener contains items related to worry and avoidance, only the total FOH score was analyzed. Therefore, the study could not determine whether higher FOH scores represented adaptive preventive behaviors, maladaptive avoidance patterns, or greater awareness of hypoglycemia risk.

### 5.2. Nursing Implications

There are subsequent implications of the findings for nursing practice. Based on the present findings and prior literature, nurses may consider assessing FOH during individualized diabetes education and follow-up, particularly among insulin-treated patients. However, the cross-sectional nature of this study necessitates further longitudinal and interventional research before recommending FOH screening as a routine practice requirement. Screening can assist nurses in differentiating adaptive concerns, which fosters vigilance, and excessive fear, which can result in avoidance or lack of quality of life. Secondly, nurses might focus on structured diabetes counseling and self-management education for the older patients (who live alone and have comorbid hypertension). Extra emphasis should be placed on the physical activity area, as it emerged as the weakest aspect of self-management in this research. The nurses are also in a good position to identify a deficiency of social support and call in the family members or community resources in instances where patients do not have day-to-day support. Lastly, DSMQ and its Arabic version can also be applicable in nursing assessment, as they offer a convenient method of determining the areas of self-management that should be reinforced and could be used to formulate individual care plans pertinent to Arabic patients with diabetes.

## Figures and Tables

**Table 1:** Characteristics of studied subjects (n = 180).

Variable	Category	n	%
Age (Mean ± SD)	36.82 ± 16.34		
Gender	Female	116	64.4
	Male	64	35.6
Marital status	Single	90	50.0
	Married	72	40.0
	Widowed	12	6.7
	Divorced	5	2.8
	Separated	1	0.6
Educational level	Bachelor’s degree	79	43.9
	Secondary school	57	31.7
	Postgraduate	18	10.0
	Primary school	14	7.8
	No formal education	12	6.7
Living status	Living with others	168	93.3
	Living alone	12	6.7
Smoking status	Non-smoker	159	88.3
	Smoker	21	11.7
Previously attended DM counseling sessions	Yes	63	35.0
	No	117	65.0
Regular exercise regimen	Yes	72	40.0
	No	108	60.0
Treatment duration	Less than 5 years	86	47.8
	5–10 years	61	33.9
	More than 10 years	33	18.3
Duration of diabetes	Less than 5 years	79	43.9
	5–10 years	63	35.0
	More than 10 years	38	21.1
Presence of hypertension	Yes	68	37.8
	No	112	62.2
High cholesterol	Yes	71	39.4
	No	109	60.6
Type of diabetes	Type 1	126	70.0
	Type 2	54	30.0
Height (Mean ± SD)	160.86 ± 13.92		
Weight (Mean ± SD)	73.18 ± 14.60		
Types of treatment	Exclusive insulin	106	58.9
	Combined with oral medication	46	25.6
	Oral medication	28	15.5

**Table 2:** Descriptive Statistics for Main Study Variables (N = 180).

Variable				Mean	SD	Min	Max
Self-management (self-care activities)				26.71	3.57	14	36
HbA1c (%)				7.95	1.85	5.0	20.0
Fear-of-hypoglycemia screening				26.10	6.69	9	45
**Domain**	**Mean**	**SD**	**Max**	**Standardized (0–10)**		**Category**	
Glucose management	8.10	2.69	15	5.40		Moderately acceptable	
Dietary control	6.87	2.11	12	5.73		Moderately acceptable	
Physical activity	4.23	1.93	8	4.70		Insufficient	
Healthcare usage	6.71	1.65	9	7.46		Moderately acceptable	
Overall item	0.80	0.68	2	-		-	
Total DSMQ	26.71	3.57	46	5.56		Moderately acceptable	

**Table 3:** Pearson Correlation Matrix with *p* Values (N = 180).

	Self-Management	HbA1c
Self-management		
HbA1c	−0.13 (0.087)	
Fear-of-hypoglycemia screener	0.49 (<0.01)	0.04 (>0.05)

**Table 4:** Multiple Linear Regression Predicting Diabetes Self-Management (N = 180).

Predictor	Reference	B	SE	95% CI	β	t	*p*	VIF
Age	—	−0.05	0.02	−0.09 to −0.01	−0.16	−2.80	0.006	1.30
Weight	—	0.03	0.02	−0.01 to 0.07	0.10	1.55	0.122	1.28
Counseling	Yes	2.60	0.68	1.25 to 3.95	0.25	3.80	<0.001	1.35
Hypertension	Yes	−2.60	0.70	−3.98 to −1.22	−0.27	−3.70	<0.001	1.42
Cholesterol	Yes	0.65	0.57	−0.47 to 1.77	0.06	1.14	0.256	1.33
Living alone	Yes	−2.80	1.08	−4.93 to −0.67	−0.20	−2.59	0.010	1.21
Exercise	Yes	0.78	0.73	−0.66 to 2.22	0.07	1.07	0.287	1.18
Type of diabetes	Type 1	0.30	0.85	−1.37 to 1.97	0.02	0.35	0.725	1.27
Treatment duration	5–10 yrs	−0.45	0.49	−1.42 to 0.52	−0.05	−0.92	0.359	1.36
Duration of diabetes	5–10 yrs	−0.65	0.43	−1.49 to 0.19	−0.08	−1.51	0.133	1.40
Marital status	Married	0.80	0.63	−0.44 to 2.04	0.08	1.27	0.206	1.25
Education	Bachelor’s	0.85	0.47	0.02 to 1.68	0.11	2.05	0.042	1.31
Treatment type	Combined	−0.52	0.58	−1.66 to 0.62	−0.05	−0.90	0.370	1.29
Fear-of-hypoglycemia screening	—	+0.27	0.04	0.19 to 0.35	+0.44	6.90	<0.001	1.05

Model fit: R^2^ = 0.501. Adjusted R^2^ = 0.430. F (14,165) = 9.5, *p* < 0.001.

## Data Availability

The data presented in this study are available on request from the corresponding author because of privacy and ethical restrictions related to participant confidentiality.

## References

[1] Fariba A., Amerzadeh M., Banazadeh M., Rashidi S., Myaneh Z.T. (2024). Fear of Hypoglycemia and Illness Perception in Type II Diabetes Patients. BMC Endocr. Disord..

[2] The Lancet (2023). Diabetes: A Defining Disease of the 21st Century. Lancet.

[3] Alves-Costa S., Nascimento G.G., de Souza B.F., Hugo F.N., Leite F.R.M., Ribeiro C.C.C. (2025). Global, Regional, and National Burden of High Sugar-Sweetened Beverages Consumption, 1990–2021, with Projections up to 2050: A Systematic Analysis for the Global Burden of Disease Study 2021. Am. J. Clin. Nutr..

[4] American Diabetes Association Professional Practice Committee (2023). 6. Glycemic Goals and Hypoglycemia: Standards of Care in Diabetes—2024. Diabetes Care.

[5] CDC (2024). Adult BMI Calculator. https://www.cdc.gov/bmi/adult-calculator/index.html.

[6] Sun H., Saeedi P., Karuranga S., Pinkepank M., Ogurtsova K., Duncan B.B., Stein C., Basit A., Chan J.C.N., Mbanya J.C. (2022). IDF Diabetes Atlas: Global, regional and country-level diabetes prevalence estimates for 2021 and projections for 2045. Diabetes Res. Clin. Pract..

[7] Ong K.L., Stafford L.K., McLaughlin S.A., Boyko E.J., Vollset S.E., Smith A.E., Dalton B.E., Duprey J., Cruz J.A., Hagins H. (2023). Global, Regional, and National Burden of Diabetes from 1990 to 2021, with Projections of Prevalence to 2050: A Systematic Analysis for the Global Burden of Disease Study 2021. Lancet.

[8] Adu M.D., Malabu U.H., Malau-Aduli A.E.O., Malau-Aduli B.S. (2019). Enablers and Barriers to Effective Diabetes Self-Management: A Multi-National Investigation. PLoS ONE.

[9] Kwame A., Petrucka P.M. (2021). A Literature-Based Study of Patient-Centered Care and Communication in Nurse-Patient Interactions: Barriers, Facilitators, and the Way Forward. BMC Nurs..

[10] Liang W., Lo S.H.S., Tola Y.O., Chow K.M. (2021). The Effectiveness of Self-Management Programmes for People with Type 2 Diabetes Receiving Insulin Injection: A Systematic Review and Meta-Analysis. Int. J. Clin. Pract..

[11] Shin K.S., Lee E.-H. (2018). Relationships of Health Literacy to Self-Care Behaviors in People with Diabetes Aged 60 and above: Empowerment as a Mediator. J. Adv. Nurs..

[12] Alyami M., Serlachius A., Mokhtar I., Broadbent E. (2019). Illness Perceptions, HbA1c, And Adherence In Type 2 Diabetes In Saudi Arabia. Patient Prefer. Adherence.

[13] Fİdan Ö., Takmak Ş., Zeyrek A.Ş., Kartal A. (2020). Patients with Type 2 Diabetes Mellitus: Obstacles in Coping. J. Nurs. Res. JNR.

[14] Im J.H.B., Escudero C., Zhang K., Choi D., Sivakumar A., Booth G.L., Sale J., Pritlove C., Advani A., Yu C.H. (2022). Perceptions and Correlates of Distress Due to the COVID-19 Pandemic and Stress Management Strategies Among Adults with Diabetes: A Mixed-Methods Study. Can. J. Diabetes.

[15] Wang M.-J., Lin H.-M., Hung L.-C., Lo Y.-T. (2020). Non-Health Outcomes Affecting Self-Care Behaviors and Medical Decision-Making Preference in Patients with Type 2 Diabetes: A Cross-Sectional Study. BMC Med. Inform. Decis. Mak..

[16] Alghrid S., Refadah O., Alharbi D., Aljuhani N., Albalawi S., Aljuhani S., Alaradi R., Yzeed A., Albalawi S., Amirthalingam P. (2023). SAT123 Treatment Satisfaction And Barriers To Diabetes Care In Patients with Uncontrolled Diabetes Mellitus: A Cross-Sectional Study. J. Endocr. Soc..

[17] Alotaibi Y.K., Al-Nowaiser N., Al Harbi T.J., Tourkmani A.M., Moharram M. (2023). Improving Type 2 Diabetes Mellitus Management in Ministry of Defense Hospitals in the Kingdom of Saudi Arabia 2018–2021. BMJ Open Qual..

[18] Khardali A., Aladwani A., Alzahrani F., Madkhali O.A., Al Qahtani S., Qadri M., Adawi M.D., Hakamy M. (2024). Exploring Patient’s Perspective of Barriers to Diabetic Medication Adherence in Jazan, Saudi Arabia, Using the Social Determinants of Health Model. SAGE Open Med..

[19] Henderson J.N., Allen K.V., Deary I.J., Frier B.M. (2003). Hypoglycaemia in Insulin-Treated Type 2 Diabetes: Frequency, Symptoms and Impaired Awareness. Diabet. Med..

[20] American Diabetes Association (2019). 6. Glycemic Targets: Standards of Medical Care in Diabetes—2020. Diabetes Care.

[21] Whitmer R.A., Gilsanz P., Quesenberry C.P., Karter A.J., Lacy M.E. (2021). Association of Type 1 Diabetes and Hypoglycemic and Hyperglycemic Events and Risk of Dementia. Neurology.

[22] Przezak A., Bielka W., Molęda P. (2022). Fear of Hypoglycemia—An Underestimated Problem. Brain Behav..

[23] Heller S.R., Peyrot M., Oates S.K., Taylor A.D. (2020). Hypoglycemia in Patient with Type 2 Diabetes Treated with Insulin: It Can Happen. BMJ Open Diabetes Res. Care.

[24] Dehesh T., Dehesh P., Shojaei S. (2020). Prevalence and Associated Factors of Anxiety and Depression Among Patients with Type 2 Diabetes in Kerman, Southern Iran. Diabetes Metab. Syndr. Obes. Targets Ther..

[25] Lambert-Obry V., Lafrance J.-P., Savoie M., Lachaine J. (2022). The Impact of Hypoglycemia on Productivity Loss and Utility in Patients with Type 2 Diabetes Treated with Insulin in Real-World Canadian Practice: Protocol for a Prospective Study. JMIR Res. Protoc..

[26] Siamashvili M., Davis S. (2021). Late Phase Completed Clinical Trials Investigating Bromocriptine Mesylate Quick Release as Treatment of Type 2 Diabetes Mellitus. Expert Opin. Pharmacother..

[27] Muneer M. (2021). Hypoglycaemia. Adv. Exp. Med. Biol..

[28] Schmitt A., Gahr A., Hermanns N., Kulzer B., Huber J., Haak T. (2013). The Diabetes Self-Management Questionnaire (DSMQ): Development and Evaluation of an Instrument to Assess Diabetes Self-Care Activities Associated with Glycaemic Control. Health Qual. Life Outcomes.

[29] Innab A., Kerari A. (2024). Validation of the Arabic Version of the Diabetes Self-Management Questionnaire in Patients with Type 2 Diabetes Mellitus: A Cross-Sectional Study. BMC Prim. Care.

[30] Al-Qahtani A.M. (2020). Frequency and Factors Associated with Inadequate Self-Care Behaviors in Patients with Type 2 Diabetes Mellitus in Najran, Saudi Arabia. Based on Diabetes Self-Management Questionnaire. Saudi Med. J..

[31] Liu J., Poon J.L., Bispham J., Perez-Nieves M., Hughes A., Chapman K., Mitchell B., Hood K., Snoek F., Fisher L. (2023). Development and validation of fear of hypoglycemia screener: Results from the T1D exchange registry. J. Patient-Rep. Outcomes.

[32] Amin S.M., Abdallah H.M.M., Zoromba M.A., Ghayth E.I., Alkubati S.A., Alqarawi N., Atta M.H.R., Alasqah I., AbouZeid N.A.A., Farghaly Abdelaliem S.M. (2026). The influence of fear of hypoglycemia and frailty on self-efficacy in diabetes management among older adults with type 2 diabetes: A cross-sectional study. Front. Psychol..

[33] Ahola A.J., Saraheimo M., Freese R., Mäkimattila S., Forsblom C., Groop P.-H., FinnDiane Study Group (2016). Fear of Hypoglycemia and Self-Management in Type 1 Diabetes. J. Clin. Transl. Endocrinol..

[34] Chrvala C.A., Sherr D., Lipman R.D. (2016). Diabetes Self-Management Education for Adults with Type 2 Diabetes Mellitus: A Systematic Review of the Effect on Glycemic Control. Patient Educ. Couns..

[35] Werfalli M.M., Kalula S.Z., Manning K., Levitt N.S. (2020). Does Social Support Effect Knowledge and Diabetes Self-Management Practices in Older Persons with Type 2 Diabetes Attending Primary Care Clinics in Cape Town, South Africa?. PLoS ONE.

[36] Baah-Nyarkoh E., Alhassan Y., Dwomoh A.K., Kretchy I.A. (2023). Medicated-Related Burden and Adherence in Patients with Co-Morbid Type 2 Diabetes Mellitus and Hypertension. Heliyon.

[37] İlhan N., Telli S., Temel B., Aştı T. (2021). Health Literacy and Diabetes Self-Care in Individuals with Type 2 Diabetes in Turkey. Prim. Care Diabetes.

[38] Adhikary K., Sarkar R., Maity S., Banerjee I., Chatterjee P., Bhattacharya K., Ahuja D., Sinha N.K., Maiti R. (2024). The underlying causes, treatment options of gut microbiota and food habits in type 2 diabetes mellitus: A narrative review. J. Basic Clin. Physiol. Pharmacol..

[39] Parua S., Hazra A., Adhikary K., Ganguly K., Ahuja D., Maiti R., Mukhopadhyay L.D., Dutta S., Panda P., Bhattacharya K. (2025). Microbial dysbiosis, obesity, and insulin resistance: Understanding gut-ovary association in polycystic ovary syndrome. Obes. Med..

